# Supplementary material for: Identification of photoperiod-induced specific miRNAs in the adrenal glands of Sunite sheep (Ovis aries)
Source: Front Vet Sci. 2022 Jul 22;9:888207. doi: 10.3389/fvets.2022.888207 (PMC9354845; doi:10.3389/fvets.2022.888207)
Supplement: Supplementary file 1 [file Data_Sheet_1.ZIP › Supplementary file/miRDeep2 file.zip!/miRDeep2 file/Novel_Structure/SPLP42A1/Novel_646.pdf]

Provisional ID	:	NC_019473.2_22575
Score total	:	16.8
Score for star read(s)	:	3.9
Score for read counts	:	8.8
Score for mfe	:	2.5
Score for randfold	:	1.6
Score for cons. seed	:	
Total read count	:	29
Mature read count	:	28
Loop read count	:	0
Star read count	:	1

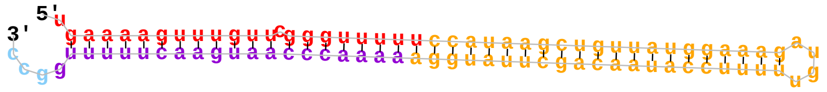

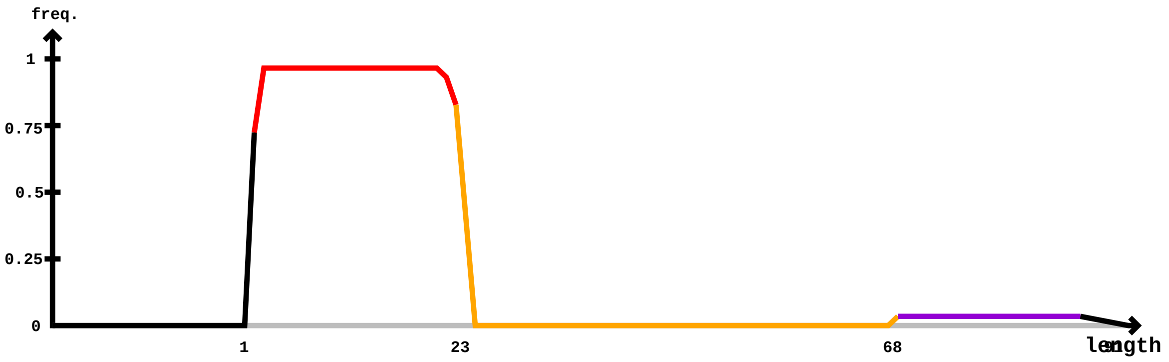


```
                           Mature                                                                 Star
5'- cccacagacacuggguuggugaaaaguuuguucggguuuuuccauaagcuguuauggaaagauguuuuuccauaacagcuuauggaaaaacccaaugaacuuuuuggccaa   -3'    obs
    cccacagacacuggguuggugaaaaguuuguucggguuuuuccauaagcuguuauggaaagauguuuuuccauaacagcuuauggaaaaacccaaugaacuuuuuggccaa          exp
    ((((.......))))(((((.(((((((((((.((((((((((((((((((((((((((((....)))))))))))))))))))))))))))))))))))))))..)))))   reads   mm   sample
    ...................ugaaaaguuuguucggguuuu.......................................................................   3       0    seq
    ...................ugaaaaguuuguucggguuuuu......................................................................   18      0    seq
    ....................Aaaaaguuuguucggguuu........................................................................   1       1    seq
    ....................gaaaaguuuguucggguuuuu......................................................................   6       0    seq
    ......................................................................................aaaacccaaugaacuuuuug.....   1       0    seq
```
